# Supplementary material for: Competencies of lower-level community health centre leaders in annual health work planning and their influence on district performance in Busoga sub-region: A retrospective study
Source: PLoS One. 2025 Jul 30;20(7):e0316055. doi: 10.1371/journal.pone.0316055 (PMC12310004; doi:10.1371/journal.pone.0316055)
Supplement: S1 Appendix — This appendix presents the structured questionnaire used to assess the managerial, technical, and interpersonal competencies of leaders at lower-level community health centres. The tool also captures key performance areas and its adaptation during budgeting and final allocations for implementation. (DOCX) [file pone.0316055.s001.docx]

**Annex**

Data collection tools

1. **District performance checklist**

| DISTRICT |  | |
| --- | --- | --- |
| SUB-DISTRICT |  | |
| Date of assessment: |  | |
| Location at the time assessment: |  | |
| Researcher: |  | |
| Facility profile: | Name: |  |
|  | Level: |  |
|  | Location: |  |
|  | Financial year: |  |

**Planning& performance tracker for the different financial years**

Planning & performance tracker

| Priority areas identified by the facility | Y | N | Priority areas adopted and included in the final plan | Y | | N |
| --- | --- | --- | --- | --- | --- | --- |
| 1. Maternal Child Health (≥ 5 indicators) | 1 | 0 | 1. Maternal Child Health | 1 | | 0 |
| 1. Health promotion, diseases prevention, sanitation and hygiene (≥ 5 indicators) | 1 | 0 | 1. Health promotion, diseases prevention, sanitation and hygiene | 1 | | 0 |
| 1. Administration (≥ 5 indicators) | 1 | 0 | 1. Administration | 1 | | 0 |
| 1. Infrastructure (≥ 3 indicators) | 1 | 0 | 1. Infrastructure | 1 | | 0 |
| 1. Resource mobilization (≥ 3 indicators) |  | 0 | 1. Resource mobilization | 1 | | 0 |
| Budget estimates per identified priority (any amount allocated during planning) | Y | N | Level of funding allocated in final plan (Evidence that amount estimated at facility is the amount in final budget) | Y | N | |
| 1. Maternal Child Health | 1 | 0 | 1. Maternal Child Health | 1 | 0 | |
| 1. Health promotion, diseases prevention, sanitation and hygiene | 1 | 0 | 1. Health promotion, diseases prevention, sanitation and hygiene | 1 | 0 | |
| 1. Administration | 1 | 0 | 1. Administration | 1 | 0 | |
| 1. Infrastructure | 1 | 0 | 1. Infrastructure | 1 | 0 | |
| 1. Resource mobilization | 1 | 0 | 1. Resource mobilization | 1 | 0 | |
| Proof of routine performance checks at facility / district level and consequent improvement in performance. | | | | Y | N | |
| 1. Quarterly performance reviews (at least 4 review meetings) | | | | 1 | 0 | |
| 1. Implementation reports (with evidence of implementation) | | | | 1 | 0 | |

1. **Competence assessment tool.**

| Facility assessment: | | | Health facility in charge | | |
| --- | --- | --- | --- | --- | --- |
|  | Y | N |  | Y | N |
| Is there a Health Unit Management Committee (HUMC)? | 1 | 0 | Ever attended planning cycle meetings organized by the district health department *(with proof of invitations)*? | 1 | 0 |
| Is the HUMC fully constituted? (At least *proof* of 6 members for HC II, and 9 members for HC III and IV). | 1 | 0 | If yes, how many times in the past five financial years? *(4 and more times is better performance – code 1)* | 1 | 0 |
| Are the HUMC members meeting the academic requirements? (At least Postgraduate, Undergraduate, UACE and UCE). | 1 | 0 | Are annual planning meetings organized at the facility *(with proof of minutes or attendance list)?* | 1 | 0 |
| Staffing level (recommended);   - 49 (for centre IV) - 19 (for centre III) - 9 (for centre II) | 1 | 0 | If yes, who participates? (*4 and more participants is better performance – code 1*)   - In charge - HUMC - Heads of units - Local leadership - Health Development Partners and CSOs | 1 | 0 |
| Who is involved in the annual planning process? (*4 and more categories of people mentioned is better performance – code 1*)   - In charge - HUMC - Heads of units / departments - Local leadership - Health Development Partners and CSOs | 1 | 0 | What tools do you use to plan? *(At least 3 and more tools used is better performance).*   - LG Planning Guidelines for the Health Sector 2019 - PHC guidelines - Public Finance Management act. - Local Government and accounting regulations. - PPDA guidelines - RBF implementation manual. - Clinical Guidelines - Others | 1 | 0 |
|  |  |  | Does the facility submit facility work plan to the district health department before the deadline? *(Consider the deadline for submission –* ***31^st^ March*** *each year).* | 1 | 0 |

Thank you for your time.
