# Supplementary material for: Competencies of lower-level community health centre leaders in annual health work planning and their influence on district performance in Busoga sub-region: A retrospective study
Source: PLoS One. 2025 Jul 30;20(7):e0316055. doi: 10.1371/journal.pone.0316055 (PMC12310004; doi:10.1371/journal.pone.0316055)
Supplement: Attachment — Submitted filename: Review of manuscript_PLOS One_by Tesfay_January17_2025.docx [file pone.0316055.s004.docx]

Dear Editor,

I hereby submit a review report of the manuscript, entitled ‘Competences of Lower Community Health Centre Leaders in Annual Health Work Plans and Its Influence On District Performance Improvement In Busoga Sub-Region: A Retrospective Study.”. I acknowledge the efforts of the authors for performing a good job and writing evidence which is highly needed. Since, it needs further amendment, I suggested to be accepted with Major revision. I believe, this manuscript will be very helpful in forwarding better evidence for decision making in rebuilding the human resource development and improving the performance of health centers.

Competences of Lower Community Health Centre Leaders In Annual Health Work Plans And Its Influence On District Performance Improvement In Busoga Sub-Region: A Retrospective Study

**Title:** is too long not catchy. Authors are advised to shorten their title to be attractive for readers.

**If authors agree they can use the alternative title that reads as**: “Competencies of frontline health facility leaders in annual work plan and its influence on district health management in Busoga Sub-Region: A Retrospective Study”

**Abstract**

**Background:** Authors need to incorporate the essential gap that makes them to conduct this research study.

**Objective:** The first statement is adequate for the objective, while authors need to move the next statement indicated in the objective to the method’s section.

**Methods:** what is the justification of authors on the adequacy of the sample of health centers for cases and controls? Authors are advised to provide valid justification for the cutoff point “12”.

Authors need to briefly describe the techniques of data collection.

**Results:** Authors are supposed to describe the number of facilities participated in the study as cases and controls.

In the methods section, Authors noticed: “There was a comparison of performance between the **worst performing (case) and best performing (control) districts** in the region according to the Annual Health Sector Performance reports from 2017/18 financial year to 2021/2022”. While in their result section they reported “the district performance in annual health work planning was not good in both the case and control groups (26.4% and 47.2% respectively).” How do authors justify for the 1.8 times fold of performance of control groups?

The result showed the 1.8 fold of best performing districts (controls) were with poor performance compared to the worst performing districts. How do authors justify the discrepancy that seems unrealistic?

**Conclusion:** authors are advised to make conclusion based on their findings.

***Keywords:*** it is better if authors limit the key words to 5

**Introduction**

The introduction section is not well organized in describing the pertinent information. It is highly dominated by the “Annual work plan concept” which is not aligned with the topic. The problem is not clearly stated. It doesn’t dictate the global experiences and the major identified gaps related to the topic.

The gap is not clearly described in the introduction section. What is the major gap that needs to be explored by authors? Is it about the leadership competency of low health center leaders or organizational annual work plan?

Authors are advised to clearly describe the pertinent components of:

1. The regional (African) experience of low health centers leaders competency in developing annual work plan and its contribution on the district performance improvement
2. The flow of their description needs to be from global to local and from general to specific.

On the second paragraph of the introduction: Authors noticed “In the Uganda Health Sector, the Annual Health Work Plan ………. performed by health centre II, Health Centre III, and ………… provide suitable performance improvement score guides and means of verification …….. for the higher local governments (2020)”. Readers might not easily understand the type of health centers except Ugandan citizens, Authors are advised to briefly describe the health system structure of the country.

**Research questions**

**Authors enquired two research questions:** 1) What is the districts’ performance in annual health work planning in the Busoga sub-region?

2) What are the competences in annual health work planning of lower health facilities

leaders that influence the performance of the districts?

However, the research questions formulated by the authors is not in line with their research title.

**Authors are advised to rephrase the research questions**: In their title authors are interested to explore the competency of low level leaders engaged at health centers and the performance improvement which is directly related to the Annual Work Planning.

**Objectives of the study**

**Specific objectives: the specific objectives are not aligned with the broad objective.**

Authors described the specific objectives: 1) To establish the districts' performance in annual work planning in the Busoga sub-region.

2) To establish the influence of competence of the lower community health centre leaders in

annual health work planning on the district performance in the Busoga sub-region.

**Authors are advised to align the specific objectives with the general objectives.**

**Study setting**

Authors stated “The study was conducted in the Busoga sub-regions of eleven (11) districts and one (1) city”, what does districts mean? Urban sub-urban or rural districts?

**Do the performance assessment standard fits to evaluate Jinga City equally with the other district? Do authors have reasons to include the City with the other district, doesn’t it create a bias to categorize city with other districts in research? For obvious reasons in cities, the competence of health workers and its managerial status is much better compared to the level of districts. Any reflections from the authors.**

**On another note, authors are advised to provide clear picture of the study setting related to the structure of the health system, availability number of health workers and the access of health institutions to the population.**

Authors used District performance and Competence in annual health work planning to measure the variables for performance. It seems many variables are measured in order to determine the status the outcome variable. **How did authors manage the analysis of multiple variables?**

**Sampling technique**

Authors noted “Multistage stratified sampling was considered to determine the number of health centre IVs, IIIs and IIs selected for the public facilities in the case and control districts where data was collected.” **Authors are advised to provide brief description about the type of health facilities in the study setting section. It is not clear for readers what the meaning of health center IV, III and II. The reason why authors used the stratified sampling technique is not clearly described in the manuscript.**

**Data collection**

Under the Document/Records Review Checklist sub heading, on the data collection section. Authors noted “A checklist **will be** a tool designed to capture information extracted from the annual health work…….” **The “will be” phrase is not acceptable at this stage; authors are advised to us the appropriate form of tense.** Similar error is shown on the statement provided under data analysis stated as “The *p*value set at 0.05 **will be used** to determine the statistical significance ……… at 95 percent confidence intervals (CI)”. “It **will derive** regression odds ratios and p-values” **Authors are advised to make correction on the highlighted phrases. Correction form of the tense is needed.**

The sub heading stated by authors “**Districts' performance in annual health work planning in Busoga sub-region**” and its content in the first paragraph doesn’t fit to the results section. Authors need to consider to move it to the methods section, because it is a descriptive narrative about the performance assessment and how health centers were graded based on their performance.

Similarly, the second paragraph that reads as “Each correct activity was scored 1, otherwise 0. A health centre that correctly did 75% of the activities was graded as good performance otherwise poor performance. Overall, the performance for each health centre level across the five financial years showed that poor performance in all the participating health centres, though control health centres performed better than the case health centres.” **It doesn’t fit to the results section. The placement of the paragraph is not appropriate. Authors are advised to revise the placement.**

**Results**

The first paragraph of the result section doesn’t seem understandable for readers. It seems confusing. **Authors are advised to present it as concise and understandable to readers. It would be good if the narrative description of the first paragraph is about the performance of the health facilities based on the standard than comparing the health facilities of cases and controls. Better to compare the health facilities of cases and controls in next paragraphs.**

On the final paragraph under the section: **Districts' performance in annual health work planning in Busoga sub-region: authors stated: “**Generally, the **control facilities** performed better than the **control facilities** in the subsequent five years”. **There is an error on the statement, authors are requested to make a correction.**

**The results of the authors need to describe the performance of the health facilities based on the annual health work planning variables. Authors are advised to provide descriptions on the work plan of processes of the major health service indicators.**

The result section would have been good if it is aligned with the methods section under the sub-heading of, **a) District performance in annual health work planning,** authors stated “In Uganda, ministry of health has set performance standards in line with maternal child health, health education and promotion, environmental determinants of health, water and sanitation, mental health and health-seeking behavior, amongst others for the lower health centre leaders.” **From this paragraph, I would expect the set of performance standards to be indicated in the result section of this manuscript. However, the result of annual health work planning in this manuscript was not integrated with the performance indicators such as: maternal and child health, health education and promotion, environmental health, water and sanitation, mental health and health-seeking behavior. Thus, authors are requested to provide brief descriptions on the strength and weakness of the categories of health service units (Maternal and child health, water and sanitation … etc) of the annual health work planning.**

On the result section of **Competences of leaders of lower community health centres in annual health work planning authors stated: “**For the 5 years, 80 percent of the health centres in the control and 83.3 percent in the case had not attended 4 or more planning cycle meetings organised by the district in the five years. Equally, most health centres did not organise annual health work planning meetings at the facility level, and less than 4 people were involved in the planning process of the work plan (43.3% for the controls and 70% for the cases).**” How do authors rely to measure competencies of leaders in the aforementioned variables? It would be difficult to consider availability of staff and organizing meetings as measurements of leaders competency. Instead, authors are advised to use a combination of variables “staff being available all the time and staff being competent, productive and responsive” according the recommendation of World Health Organization (WHO).**

**Table IV.** Performance of the health facilities in Busoga sub-region across 5 fiscal years period:

**The table shows only percentages; authors are requested to include the frequencies beside the percentages for clarity.**

**Zero count is not clear, so authors are advised to provide brief information.**

**The** HCIV performance in table IV is reported in FY 2021/22 is reported 9.1% while in 2020/2021 it was 45.5%, authors need to provide the possible and potential reasons for the highly declining performance of this particular institution. Since evidence generation is useful for policy making decisions, authors should recommend possible solutions for this particular institution.

**Table 5:** Showing the competence of the health facilities

There a HUMC

Yes

No

How do authors decide to measure competency of the health facilities with one-time availability of staff? Authors didn’t show the five years’ data on table 5. **Authors are advised to clearly indicate the five-year data in terms of health facility management committee availability.** The table needs to be again developed in a way to show the five years data.

Staffing level meets the standards for the facility

Yes

No

Authors didn’t describe about the staffing level of competency to meet the standards of the facility. **Authors are advised to indicate either standard or operational definitions that describe the staffing level to meet the standard of the facility.**

Except one variable stated as **“**Tool used for developing annual health work plan” **in table 5, authors didn’t illustrate a specific table related to availability of manuals, guidelines and standard operating procedures that helps to staff members in performing effectively. Authors seem to overlook manuals and guidelines in improving the performance and competency of health workers in the health facilities. So, authors are requested to provide their reflections in this regard.**

**Table 6:** Influence of competence of the lower health centre leaders in annual health work planning on the performance in the Busoga sub-region 2017/18-2021/22

Authors tried to calculate the Chi square and P value to see the competency of low health center leaders.

Due to the nature of susceptibility to selection bias, retrospective case–control studies need to be carefully handled using the best data analysis method that fits to the type of data collected. I am surprised why authors didn’t raise these issues as concern. The way the calculation is made is not clear**. It would be difficult to understand if the frequency of each variable is not indicated on the table along the other column items. So, authors are advised to revise the statistical test, calculating odds ratio might be also another option, it can also show the confidence interval. Besides, the table should be reformulated consisting the frequency of each variable.**

**Discussion**

**This manuscript seems an evolving, I didn’t see similar manuscripts before. The discussion seems fine, but still needs further work. If the authors tried to go back to their data and accordingly incorporate the comments provided on the results section I hope additional similarities and variations of the new results could emerge on the discussion section.**

**Authors mentioned very important terminologies related to leaders competency in the middle of theeir manuscript such as: productivity, efficiency, effectiveness, and quality. Why do authors brought these terminologies in their manuscript, while not indicated in their findings? Could authors recheck their data if it captures more information?**

**Conclusion: the conclusion seems overriding the findings of the study. Authors stated “**Health centres that meet **staffing standards**, actively participate in district planning cycles, and utilize a diverse set of implementation manuals for work planning are better positioned to contribute to district-wide health improvements.**” Though the statement is true, but the highlighted phrase “staffing standards” was not appropriately defined and not comprehensively applied in the data collection tool. The data collection tool lacks comprehensiveness. So authors need to balance the intensity of the statement on the conclusion towards the actual findings of the data. Otherwise, it would be confusing for readers.**

**Recommendation**

**Authors need to check the alignment of the recommendations with their findings.**

**Decision: Major revision**
