# Supplementary material for: Competencies of lower-level community health centre leaders in annual health work planning and their influence on district performance in Busoga sub-region: A retrospective study
Source: PLoS One. 2025 Jul 30;20(7):e0316055. doi: 10.1371/journal.pone.0316055 (PMC12310004; doi:10.1371/journal.pone.0316055)
Supplement: Attachment — Submitted filename: RESPONSE TO REVIEWERS..docx [file pone.0316055.s007.docx]

RESPONSE TO REVIEWERS’ COMMENTS:

| SECTION | Comments | Response | Page number |
| --- | --- | --- | --- |
| Title | Reviewer#1: The word competence being a noun, would sound proper if it was ‘’COMPETENCES IN ANNUAL WORK PLANNING’’ than ‘’COMPETENCE IN WORK PLANS’’. | The word competencies is a better one to use in this case (academic writing). | Page 1 |
|  | Reviewer #2: Title: is too long not catchy. Authors are advised to shorten their title to be attractive for readers.  If authors agree they can use the alternative title that reads as: “Competencies of frontline health facility leaders in annual work plan and its influence on district health management in Busoga Sub-Region: A Retrospective Study.” | Competencies of frontline health facility leaders in annual work plan is catchy. However, it will consider leaders in all levels of health centres, yet this study looked at lower level (centre IV and below) leaders where annual health work planning was not or partially done.  The title has been slightly changed and in line with the suggested ideas by the reviewer as;  “Competencies of Lower-Level Community Health Centre Leaders in Annual Health Work Planning and Their Influence on District Performance in Busoga Sub-Region: A Retrospective Study” | Page 1 |
| Abstract | Reviewer #2: Background: Authors need to incorporate the essential gap that makes them to conduct this research study. | The gap has been incorporated. | Page 1 |
|  | Reviewer #2: Objective: The first statement is adequate for the objective, while authors need to move the next statement indicated in the objective to the method’s section. | Next statement was deleted under objective and transferred to methodology. | Page 1 |
|  | Reviewer #2: **Methods:** what is the justification of authors on the adequacy of the sample of health centers for cases and controls? Authors are advised to provide valid justification for the cutoff point “12”.  Authors need to briefly describe the techniques of data collection | The sample of health centres for cases and controls are adequate in reference to previous studies like Muwanguzi, et al. (2020) study on the effectiveness of training of health unit management committee (HUMC) members, who quoted Schelssman’s (1982) formula to determine the sample size of health centres. Also, geographically, health centres in the region are scattered and resource constraints has to be keenly considered.  Techniques of data collection were structured interviews of leaders by research assistants (at least 5 in each centre) and documentary review (especially the annual work plan) for reviewing certain indicators suggested by the study, but also to verify answers provided by respondents. | Page 8  Page 9 |
|  | Reviewer #2: **Conclusion:** authors are advised to make conclusion based on their findings.  ***Keywords:*** it is better if authors limit the key words to 5 | Conclusion has been modified according to findings.  Key words have been reduced to 5. | Page 2 & 16  Page 2 |
| Introduction | Reviewer #2: What is the major gap that needs to be explored by authors? Is it about the leadership competency of low health center leaders or organizational annual work plan? | There is still a gap in terms of competence in making annual work plans among lower facilities leaders towards making effective annual work plans.  This has been reflected in the main document | Page 4 |
|  | Reviewer #2: Authors are advised to clearly describe the pertinent components of:   1. The regional (African) experience of low health centers leader’s competency in developing annual work plan and its contribution on the district performance improvement 2. The flow of their description needs to be from global to local and from general to specific. | Some studies from Ghana; Malawi and Uganda have been cited and included.  However, there’s no much literature on this exact subject matter. | Page 3 and 4 |
|  | Reviewer #2: Readers might not easily understand the type of health centers except Ugandan citizens, Authors are advised to briefly describe the health system structure of the country. | This has been addressed and the health system structure included. | Page 2 |
|  | Reviewer #2: **Objectives of the study**  **Specific objectives: the specific objectives are not aligned with the broad objective.**  Authors described the specific objectives: 1) To establish the districts' performance in annual work planning in the Busoga sub-region.  2) To establish the influence of competence of the lower community health centre leaders in  annual health work planning on the district performance in the Busoga sub-region.  **Authors are advised to align the specific objectives with the general objectives.** | The objectives have been re phrased as;  To assess the level of performance of districts in annual health work planning in Busoga region.  To establish the competencies of lower level community health centre leaders in annual work planning that influence district performance in Busoga region. | Page 4 |
| Methodology | Reviewer #1. Document/ records review checklist, Validity of instruments, Reliability of the instruments and Data analysis,  Use the right tense (past) and grammar in this section. | The tenses have been changed to past tenses and the grammar corrected. | Page 9 |
|  | Reviewer #1. Dependent variable: The study considers the competences of the leaders of the health centers but in actual sense, it is the competences of the health facilities. The researcher may need to explicitly state the assumption that the health facility competences are synonymous with the leaders’ competences. | The study considered competencies of lower level community health centre leaders in the health centres.  The dependent variable for this study is district performance. | Page 5 |
|  | Reviewer #1. Methodology: This is a case control study according to the researcher. The controls however do not meet the criteria set out by the study. In fact, the control group participants are cases by definition. There has to be a justification for this decision by the researcher or else this is a major methodological flaw. It would have been better if the researchers considered a retrospective cohort of under performers (more so after noticing that the control group criteria was out of reach). In this case an independent t-test would have been a good choice at analysis. | This study considered performance of districts in order to come up with the two groups as cases for poor performance and controls for good performance. There was no intervention(s) in both the case and control groups. It is one reason the study investigated whether competencies of lower-level community health center leaders in annual health work planning influenced district performance in both groups.  *Perhaps, this study can inform in future for a cohort study with a defined intervention to be followed.*  In this study, the controls were districts with performance above the national average (NA) referred to as good performing districts for at least four years in the period 2017/18 to 2021/22 and these included Jinja, Iganga, Kamuli and Kaliro (see table 1). Then, the cases were districts with performance below the national average (NA) referred to as poor performing districts for at least three years in the period 2017/18 and included Namutumba, Namayingo, Mayuge and Luuka.  Linking competencies of leaders to the district performance, Odds Ratio quantifies how much likely are competent leaders perform better compared to incompetent leaders. This is well stated in the methodology under data analysis. | Page 6  Page 9 |
|  | Reviewer #1. Conclusion: The study makes bold conclusions on the backdrop of a methodological error. | Methodological error has been corrected and clarified. |  |
|  | Reviewer #1. Readability: The manuscript needs to be trimmed to a more concise version. | The manuscript has been trimmed | All pages |
| Results: | Reviewer #2. Authors are supposed to describe the number of facilities participated in the study as cases and controls. | Description of facilities has been done. | Page 7 -8 |
|  | Reviewer #2. In the methods section, Authors noticed: “There was a comparison of performance between the worst performing (case) and best performing (control) districts in the region according to the Annual Health Sector Performance reports from 2017/18 financial year to 2021/2022”. While in their result section they reported “the district performance in annual health work planning was not good in both the case and control groups (26.4% and 47.2% respectively).” How do authors justify for the 1.8 times fold of performance of control groups? | The poor performing districts and good performing districts were selected based on the national average score which the study based on to categorize the districts into cases and controls in the region. However, this did not guarantee how the two groups (cases and controls) can perform in terms of competences of the lower-level health centre leaders.  The average score for the priority areas was considered and 75% and above score was for good performing districts. However, averagely, the districts as cases and controls did not meet 75%, hence poor performance in all groups. | Page 6 |
|  | Reviewer #2. The result showed the 1.8 fold of best performing districts (controls) were with poor performance compared to the worst performing districts. How do authors justify the discrepancy that seems unrealistic? | That is what we discovered with our findings, that competencies of leaders did not influence the good performance of districts. | Page 11 |
| Study area | Reviewer #2. Authors stated “The study was conducted in the Busoga sub-regions of eleven (11) districts and one (1) city”, what does districts mean? Urban sub-urban or rural districts? | Districts mean rural districts. It has been modified in the methodology. | Page 6 |
|  | Reviewer #2. Do the performance assessment standard fits to evaluate Jinja City equally with the other district? Do authors have reasons to include the City with the other district, doesn’t it create a bias to categorize city with other districts in research? For obvious reasons in cities, the competence of health workers and its managerial status is much better compared to the level of districts. Any reflections from the authors. | Constitutionally, in Uganda, a city is equivalent to a district. Therefore, in terms of stating districts making up Busoga region, we could not miss Jinja city.  However, the performance standard fits in the rural districts than the urban district. Jinja city being an urban would not meet the selection criteria. | Page 7 |
|  | Reviewer #2. On another note, authors are advised to provide clear picture of the study setting related to the structure of the health system, availability number of health workers and the access of health institutions to the population. | It has been addressed in the introduction and methodology section. |  |
|  | Reviewer #2. Authors used District performance and Competence in annual health work planning to measure the variables for performance. It seems many variables are measured in order to determine the status the outcome variable. How did authors manage the analysis of multiple variables? | The authors aggregated multiple indicators into a single performance score by assigning weights to each indicator based on its importance. This was done through summation and averaging. District performance was measured as a good one when three quarters (75%) of the priority areas identified in the facility work plan were realized. It was guided by the national average score which ranged from approximately 65 to 74 percent (approx. ^3^/_4_ of the performance mark. | Page 6 |
| Sampling technique | Reviewer #2. Authors are advised to provide brief description about the type of health facilities in the study setting section. It is not clear for readers what the meaning of health center IV, III and II. The reason why authors used the stratified sampling technique is not clearly described in the manuscript. | It has been handled in the introduction and methodology. | Many pages |
| Data collection | Reviewer #2. Under the Document/Records Review Checklist sub heading, on the data collection section. Authors noted “A checklist will be a tool designed to capture information extracted from the annual health work…….” The “will be” phrase is not acceptable at this stage; authors are advised to us the appropriate form of tense. Similar error is shown on the statement provided under data analysis stated as “The pvalue set at 0.05 will be used to determine the statistical significance ……… at 95 percent confidence intervals (CI)”. “It will derive regression odds ratios and p-values” Authors are advised to make correction on the highlighted phrases. Correction form of the tense is needed. | Tenses have been corrected | Pages 7 – 8 |
| Results | Reviewer #2. The sub heading stated by authors “Districts' performance in annual health work planning in Busoga sub-region” and its content in the first paragraph doesn’t fit to the results section. Authors need to consider to move it to the methods section, because it is a descriptive narrative about the performance assessment and how health centers were graded based on their performance. | Paragraph shifted to methodology | Page 5 |
|  | Reviewer #2. Similarly, the second paragraph that reads as “Each correct activity was scored 1, otherwise 0. A health centre that correctly did 75% of the activities was graded as good performance otherwise poor performance. Overall, the performance for each health centre level across the five financial years showed that poor performance in all the participating health centres, though control health centres performed better than the case health centres.” It doesn’t fit to the results section. The placement of the paragraph is not appropriate. Authors are advised to revise the placement. | The paragraph has been rephrased and shifted to methodology | Page 5 |
|  | Reviewer #2. The first paragraph of the result section doesn’t seem understandable for readers. It seems confusing. Authors are advised to present it as concise and understandable to readers. It would be good if the narrative description of the first paragraph is about the performance of the health facilities based on the standard than comparing the health facilities of cases and controls. Better to compare the health facilities of cases and controls in next paragraphs. | This has been done. | Page 12 |
|  | Reviewer #2. On the final paragraph under the section: Districts' performance in annual health work planning in Busoga sub-region: authors stated: “Generally, the control facilities performed better than the control facilities in the subsequent five years”. There is an error on the statement, authors are requested to make a correction. | Error has been corrected. |  |
|  | Reviewer #2. The results of the authors need to describe the performance of the health facilities based on the annual health work planning variables. Authors are advised to provide descriptions on the work plan of processes of the major health service indicators. | This has been corrected. |  |
|  | Reviewer #2. The result section would have been good if it is aligned with the methods section under the sub-heading of, a) District performance in annual health work planning, authors stated “In Uganda, ministry of health has set performance standards in line with maternal child health, health education and promotion, environmental determinants of health, water and sanitation, mental health and health-seeking behavior, amongst others for the lower health centre leaders.” From this paragraph, I would expect the set of performance standards to be indicated in the result section of this manuscript. However, the result of annual health work planning in this manuscript was not integrated with the performance indicators such as: maternal and child health, health education and promotion, environmental health, water and sanitation, mental health and health-seeking behavior. Thus, authors are requested to provide brief descriptions on the strength and weakness of the categories of health service units (Maternal and child health, water and sanitation … etc) of the annual health work planning. | This has been summarized into good and poor performance of districts in the control and cases as districts respectively. Variables under performance of the district have guided in understanding the general performance of districts in annual health work planning. |  |
|  | Reviewer #2. On the result section of Competences of leaders of lower community health centres in annual health work planning authors stated: “For the 5 years, 80 percent of the health centres in the control and 83.3 percent in the case had not attended 4 or more planning cycle meetings organised by the district in the five years. Equally, most health centres did not organise annual health work planning meetings at the facility level, and less than 4 people were involved in the planning process of the work plan (43.3% for the controls and 70% for the cases).” How do authors rely to measure competencies of leaders in the aforementioned variables? It would be difficult to consider availability of staff and organizing meetings as measurements of leaders competency. Instead, authors are advised to use a combination of variables “staff being available all the time and staff being competent, productive and responsive” according the recommendation of World Health Organization (WHO). | Words have been rephrased to suit competencies as defined by WHO.  In this study, competencies of lower-level health centre leaders were defined by their ability to engage community stakeholders like the mandated HUMC to participate in annual health work planning. Equally, managerial and administrative competencies were also considered. For instance, leaders should exercise the skills of using the limited staff for results (work planning) through motivation, close supervision or invitations to participate in the annual work planning. There many other competencies like problem-solving and decision-making competencies where leaders become innovative in the planning meetings, and technical and professional competencies were health centre leaders are able to identify and understand the national policies and guidelines used in the annual health work planning. | Page 7 |
|  | Reviewer #2. Table IV. Performance of the health facilities in Busoga sub-region across 5 fiscal years period: The table shows only percentages; authors are requested to include the frequencies beside the percentages for clarity. | Table has been deleted. It seems to be irrelevant. |  |
|  | Reviewer #2. Zero count is not clear, so authors are advised to provide brief information. The HCIV performance in table IV is reported in FY 2021/22 is reported 9.1% while in 2020/2021 it was 45.5%, authors need to provide the possible and potential reasons for the highly declining performance of this particular institution. Since evidence generation is useful for policy making decisions, authors should recommend possible solutions for this particular institution. | With the modification of the research questions and objectives, this information was found irrelevant and therefore deleted. |  |
|  | Reviewer #2. Table 5: Showing the competence of the health facilities There a HUMC Yes No How do authors decide to measure competency of the health facilities with one-time availability of staff? Authors didn’t show the five years’ data on table 5. Authors are advised to clearly indicate the five-year data in terms of health facility management committee availability. The table needs to be again developed in a way to show the five years data. | It has been modified accordingly. |  |
|  | Reviewer #2. Staffing level meets the standards for the facility Yes No Authors didn’t describe about the staffing level of competency to meet the standards of the facility. Authors are advised to indicate either standard or operational definitions that describe the staffing level to meet the standard of the facility. | It has been modified |  |
|  | Reviewer #2. Except one variable stated as “Tool used for developing annual health work plan” in table 5, authors didn’t illustrate a specific table related to availability of manuals, guidelines and standard operating procedures that helps to staff members in performing effectively. Authors seem to overlook manuals and guidelines in improving the performance and competency of health workers in the health facilities. So, authors are requested to provide their reflections in this regard. | It has been modified |  |
|  | Reviewer #2. Table 6: Influence of competence of the lower health centre leaders in annual health work planning on the performance in the Busoga sub-region 2017/18-2021/22  Authors tried to calculate the Chi square and P value to see the competency of low health center leaders. Due to the nature of susceptibility to selection bias, retrospective case–control studies need to be carefully handled using the best data analysis method that fits to the type of data collected. I am surprised why authors didn’t raise these issues as concern. The way the calculation is made is not clear. It would be difficult to understand if the frequency of each variable is not indicated on the table along the other column items. So, authors are advised to revise the statistical test, calculating odds ratio might be also another option, it can also show the confidence interval. Besides, the table should be reformulated consisting the frequency of each variable. | The result section has been modified as per the guidance of the reviewer. |  |
| Discussion | Reviewer #2. This manuscript seems an evolving, I didn’t see similar manuscripts before. The discussion seems fine, but still needs further work. If the authors tried to go back to their data and accordingly incorporate the comments provided on the results section I hope additional similarities and variations of the new results could emerge on the discussion section. | We have modified whole manuscript especially the methodology and results. |  |
|  | Reviewer #2. Authors mentioned very important terminologies related to leaders competency in the middle of their manuscript such as: productivity, efficiency, effectiveness, and quality. Why authors do brought these terminologies in their manuscript, while not indicated in their findings? Could authors recheck their data if it captures more information? | Terminologies were captured for purposes of the reader pick the concept very well. It was not necessary in findings. Document has been improved and modified. |  |
| Conclusion: | Reviewer #2. The conclusion seems overriding the findings of the study. Authors stated “Health centres that meet staffing standards, actively participate in district planning cycles, and utilize a diverse set of implementation manuals for work planning are better positioned to contribute to district-wide health improvements.” Though the statement is true, but the highlighted phrase “staffing standards” was not appropriately defined and not comprehensively applied in the data collection tool. The data collection tool lacks comprehensiveness. So authors need to balance the intensity of the statement on the conclusion towards the actual findings of the data. Otherwise, it would be confusing for readers. | It has been corrected. |  |
| Recommendations | Reviewer #2. Authors need to check the alignment of the recommendations with their findings | This has been checked. |  |
