# Supplementary material for: Competencies of lower-level community health centre leaders in annual health work planning and their influence on district performance in Busoga sub-region: A retrospective study
Source: PLoS One. 2025 Jul 30;20(7):e0316055. doi: 10.1371/journal.pone.0316055 (PMC12310004; doi:10.1371/journal.pone.0316055)
Supplement: Attachment — Submitted filename: Re-review of Manuscript_March 31st_ACcepted decision.docx [file pone.0316055.s006.docx]

March 31^st^, 2025

**Dear Editor,**

I hereby submit revised report of the manuscript, entitled “Competencies of Lower-Level Community Health Centre Leaders in Annual Health Work Planning and Their Influence on District Performance in Busoga SubRegion: A Retrospective Study”, after reading and checking all the revised manuscript. I acknowledge the efforts of the authors for revising the manuscript and developed a good evidence which is highly needed. Now, authors have incorporated all comments and I can say the manuscript is now in better position. I suggested to be accepted immediate after correction made for the underneath comments by authors. I believe, this manuscript will be very helpful in forwarding better evidence for decision making in rebuilding the human resource development and improving the performance of health centers.

**Revised title:** “Competencies of Lower-Level Community Health Centre Leaders in Annual Health Work Planning and Their Influence on District Performance in Busoga SubRegion: A Retrospective Study”

Authors need to revise the comments below.

Authors wrote the specific objectives with a verb that is not explanatory. **“To establish”**

Specific objectives 1) **To establish** the districts' performance in annual work planning in the Busoga sub-region. 2) **To establish** the influence of competence of the lower community health centre leaders in annual health work planning on the district performance in the Busoga sub-region.

**The phrase needs to be replaced with an action verb. “To assess” or with another action verb that fits with the objective statement.**
