# Supplementary material for: Competencies of lower-level community health centre leaders in annual health work planning and their influence on district performance in Busoga sub-region: A retrospective study
Source: PLoS One. 2025 Jul 30;20(7):e0316055. doi: 10.1371/journal.pone.0316055 (PMC12310004; doi:10.1371/journal.pone.0316055)
Supplement: Attachment — Submitted filename: Response to Reviwers.docx [file pone.0316055.s008.docx]

| Comments | Response | Page number |
| --- | --- | --- |
| Reviewer #1: The authors have satisfactorily addressed most of the comments. However, one issue is still unresolved. The authors refer to this as a case control study in their methods and results. The authors clearly defined their criteria for cases and controls using a national average of 75% in performance; implying that the scores above 75% good performance (controls) and the performance scores below 75% poor performance (cases). The authors admit that they didn't get the outcome that meets the above definition. In fact all the sampled population fall under the category of cases and therefore doesn't give a good comparison for this study. "Overall, the performance of districts in annual health work planning was poor in both cases and controls, at 26.4% and 47.2%, respectively". If this statement is correct then this ceases to be a case-control study. I would suggest that the authors simply call this a retrospective cohort and the regression analysis done still conveys the same message. Otherwise, this would be misleading to the reading community. Once the above have been addressed, the paper should be ready for publication. | As authors, we praise the reviewers for appreciating our efforts and keenly guiding us to fine-tune our work.  Our original design intent is a case-control study and a retrospective case-control study, which is defined as; cases – districts that performed poorly according to the national average score in annual health sector performance report for the past five years and controls – districts that performed well (above national average).  The authors believed something *(exposure)* must have contributed to the better or poor performance of the districts in the past years which perhaps was not reported in the national health sector performance report. That is when authors thought about competencies of health centre leaders in annual work plan development (as exposure) in the cases and controls*. Therefore, the cases and controls had similar exposure (competencies of health centre leaders in work plan development). So, the exposure (competencies of leaders) doesn’t differentiate the groups.*  The *outcome was district performance in the annual health work plan development*, which was low for the cases and controls, at 26.4% and 47.2%, respectively. This means both groups had weaknesses / limited competencies of leaders in annual health work plan development, but it does not change the fact that they were originally categorized by overall district performance (as per the annual health sector performance reports).  A null finding is still valid and useful — it tells us that something *other than leader competence in annual health work plan development* likely explains district performance differences.  Shifting from a case-control to a cohort study is definitely possible, but it requires reframing the study design, group selection, and sometimes data structure. *For example it will require selecting districts based on exposure (competence level of health center leaders in annual work plan development).* That is, districts with competent health center leaders will be the exposed cohort and those with incompetent ones as unexposed cohort. Then, next will be to assess district performance. We shall be interested in knowing if *districts with competent leaders perform better over time compared to those with less competent ones.*  Even for a retrospective study, cohort study will require to start with exposure (that is, competent leaders vs. incompetent leaders) and followed up in the groups of exposed cohort and unexposed cohort respectively to see what outcomes arise.  *The case-control study did not compare competent and incompetent leaders in annual health work plan development.*  *Lastly, analysis is most likely to change. Retrospective cohort study may change to Risk Ratios instead of Odds ratios for the case-control.*  If the explanation above is convincing, we request the study remains a valid retrospective case-control study despite of the finding that the hypothesized exposure didn’t explain the observed differences. It’s still important evidence.  Otherwise a cohort study will require major changes. |  |
| Reviewer #2: Authors wrote the specific objectives with a verb that is not explanatory. “To establish” Specific objectives 1) To establish the districts' performance in annual work planning in the Busoga sub-region. 2) To establish the influence of competence of the lower community health centre leaders in annual health work planning on the district performance in the Busoga sub-region.  The phrase needs to be replaced with an action verb. “To assess” or with another action verb that fits with the objective statement. | The verb (establish) which is not explanatory was changed to active verb (assess) for both objectives as;   1) To assess the districts' performance in annual work planning in the Busoga sub-region. 2) To assess the influence of competence of the lower community health centre leaders in annual health work planning on the district performance in the Busoga sub-region. | Page 4 |
| Reference list | Referencing was made as per the PLOS ONE guidelines. | Page 20 / 21 |

RESPONSE TO COMMENTS OF THE REVIEWERS
